# Prevalence and Risk Factors of Anaemia among Orang Asli Children in Malaysia: A Scoping Review

**DOI:** 10.3390/nu15061493

**Published:** 2023-03-20

**Authors:** Muhamad Khairul Nazrin Khalil, Mohamad Aznuddin Abd Razak, Fatin Athirah Tahir, Norhafizah Sahril, Nik Adilah Shahein, Muhammad Solihin Rezali, Muhammad Azri Adam Adnan, Siaw Hun Liew, Nor’ain Ab Wahab, Norliza Shamsuddin, Mohd Shaiful Azlan Kassim

**Affiliations:** Centre for Family Health Research, Institute for Public Health, Ministry of Health, Shah Alam 40170, Malaysia

**Keywords:** anaemia, indigenous, Orang Asli, children, Malaysia

## Abstract

Background: Anaemia continues to be a global public health burden affecting all age groups, particularly children. Indigenous people, including the Orang Asli (OA) population in Malaysia, are at risk of anaemia due to the vast disparities in social determinants of health in their population compared to the non-indigenous population. Objectives: This review aimed to identify the prevalence and risk factors of anaemia among OA children in Malaysia and analyse the knowledge gaps. Methods: A systematic search was conducted in PubMed, Cochrane Library, Scopus and Google Scholar databases. This review followed Preferred Reporting Items for Systematic Reviews and Meta-Analyses extension for scoping reviews (PRISMA-ScR) guidelines. Results: This review identified six studies involving the participation of OA children from eight subtribes residing in Peninsular Malaysia. The overall prevalence of anaemia among OA children ranged from 21.6 to 80.0%, with iron deficiency anaemia prevalence at 34.0%. The risk factors of anaemia among OA children reported from one study in this review were being younger than ten years old children (AOR 2.11 (95% CI 1.23, 3.63)) and moderate to heavy Ascaris infections (AOR 2.05 (95% CI 1.12, 3.76)). There was no data from OA children from certain age groups and subtribes. Additionally, there is a paucity of data on risk factors for anaemia among OA children from the currently available evidence. Conclusion: The prevalence of anaemia among OA children poses a moderate to severe public health concern. Therefore, more comprehensive studies in the future are needed to address the gaps identified in this review, primarily regarding anaemia risk factors. This data would encourage policymakers in devising effective national prevention strategies to improve morbidity and mortality among OA children in the future.

## 1. Introduction

Anaemia continues to be a global burden of chronic diseases affecting about a quarter of the world population, with the most significant number of affected individuals in the Southeast Asian region (315 million) [1]. Anaemia is a condition in which the body’s levels of circulating red blood cells or haemoglobin fall below what is considered normal for a particular population group [2]. There are many aetiologies of anaemia, with iron deficiency being the most common cause in nearly all global regions, according to a systematic analysis of global anaemia [3]. Other causes include other micronutrient deficiencies (e.g., vitamin A, vitamin B12, folate, riboflavin), genetic haemoglobin disorders (e.g., thalassemia, sickle cell disease), infectious diseases (e.g., parasitic infections, malaria, HIV, tuberculosis) and chronic diseases [4]. Due to the disease’s various aetiologies and the non-specific nature of its symptoms, diagnosing anaemia can be difficult [5].

Anaemia can happen at any stage of life, but it is more common in children and women who are of reproductive age. It is estimated that around 600 million preschool and school-aged children are anaemic across the globe [6]. In Malaysia, the latest data from National Health Morbidity Survey (NMHS) in 2019 shows that the overall prevalence of anaemia for the population aged 15 and above was 21.3% [7]. In another study conducted among preschool and school-aged children (6 months to 12 years old) in 2011, the prevalence of anaemia and iron deficiency anaemia (IDA) was reported at 6.6% and 4.4%, respectively [8]. Numerous studies have shown that untreated anaemia in children can have a variety of long-term negative impacts on them, including the impact on their physical development, cognitive development, psychomotor development, auditory function, visual function and immunity [9,10,11].

Indigenous populations around the world are affected by anaemia, yet they are frequently unrecognised and undocumented. The term “indigenous people” in Malaysia refers to ethnic minorities made up of a variety of ethnolinguistic groups residing in Peninsular Malaysia as well as the east Malaysian states of Sarawak and Sabah [12]. The indigenous people that live in Peninsular Malaysia are known as Orang Asli (OA), while the rest are known as the natives of Sarawak or Sabah [12]. According to the Department of Orang Asli Development (JAKOA), there was a total number of 206,777 OA people living in nine states of Peninsular Malaysia as of 31st of December 2020 [13]. The OA people include 18 ethnic subgroups classified into three main tribes, namely Negrito (Bateq, Kensiu, Kintak, Lanoh, Jahai and Medriq), Senoi (Temiar, Che Wong, Mahmeri, Jahut, Semoq Beri and Semai) and Proto-Malay (Temuan, Kuala, Kanaq, Seletar, Jakun and Semelai) [14].

Recent data nationwide showed that the prevalence of anaemia among the indigenous population is higher than the general population, affecting primarily children in most parts of the world [15]. There has been an increasing number of studies reporting on anaemia among the indigenous population over the past two decades [16,17,18]. Earlier studies published over a decade ago reported that the prevalence of anaemia among OA children in Malaysia was at a severe level ranging from 41.5% to 48.5% [15,19,20]. Thus far, the available data on anaemia among OA children in Malaysia are still limited and are obtained from small-scale studies involving only certain OA tribes.

Therefore, this study aimed to summarise the existing data on anaemia among OA children to identify the prevalence and risk factors of anaemia among OA children and to analyse the research gaps in this research area. Results from this study can be utilised to inform policymakers regarding the magnitude of anaemia among OA children to devise effective strategies to improve morbidity and mortality in this population. In addition, future research could aim for more valuable and useful data by focusing on research that could fill the gaps in this area.

## 2. Materials and Methods

This review of anaemia among OA children in Malaysia was conducted following the framework outlined by Arksey and O’Malley [21] and reported following the Preferred Reporting Items for Systematic Reviews and Meta-Analyses extension for scoping reviews (PRISMA-ScR) checklist [22] (Available from Supplementary Table S1). This review is registered with National Medical Research Register (NMRR) Malaysia (NMRR-20-1553-55891). The authors prepared a review protocol before the study commencement; however, it was not published anywhere.

### 2.1. Search Strategies

A systematic search was performed using different databases and other registries (Google, Google Scholar, reference lists of relevant articles) to identify eligible studies published during the last ten years up to the 30th of April 2022. Electronic databases searched were PubMed, Scopus, Cochrane Library and Google Scholar. The search terms used in this study based on the following keywords: (1) population (Orang Asli children, Negrito children, Senoi children and Melayu Proto children), (2) outcome (anaemia MeSH term and iron-deficiency anaemia) and (3) location (Malaysia). A detailed search strategy performed by the authors in this review is available in (Available from Supplementary Figure S1).

### 2.2. Eligibility Criteria

This review included published studies on the prevalence of anaemia among OA children in Malaysia. The inclusion criteria were (1) primary studies reported on the prevalence and/or risk factors of anaemia among OA children, (2) studies that include OA children with other population(s) (i.e., children of different ethnicity or adults), taking into consideration that the data for OA children were reported separately from others, (3) conducted in Malaysia, (4) published between the years 2012 and 2022 and (5) published in the Malay or English language. There was no restriction in terms of sample size. The term child was defined according to Conventions on the Rights of the Child (CRC) [23], which refers to individuals below 18 years of age, while the term anaemia was defined according to the criteria outlined by the World Health Organization (WHO) [2]. The primary outcome of this study was the anaemia status among OA children according to WHO criteria.

### 2.3. Selection Process

Duplicate studies obtained from the four databases were removed. The authors (M.K.N.K., M.A.A.R., F.A.T., N.S. (Norhafizah Sahril), N.A.S., M.S.R., M.A.A.A., S.H.L., N.A.W. and N.S. (Norliza Shamsuddin)), working in pairs, performed title and/or abstract screening independently for potentially eligible studies and removed irrelevant studies. Then, M.K.N.K. and M.A.R. independently reviewed the full texts of the shortlisted studies for inclusion and showed reasons for excluding any study. Any disagreement between the two authors was resolved via consultation with another author (F.A.T.) when necessary.

### 2.4. Data Extraction, Synthesis and Charting

M.K.N.K. and M.A.A.R. independently extracted the data from the included studies using a standardised electronic data extraction form. The form was developed in a Microsoft Excel spreadsheet to extract relevant information from each study. The relevant information extracted were the characteristics of the study (titles, author of the studies/year of publication and study designs), population characteristics (OA tribe, age range, sample size and sampling frame) and the outcome (prevalence and risk factors of anaemia among OA children). All data extracted was checked by F.A.T. for accuracy. Then, the data were descriptively reported according to study characteristics, the prevalence of anaemia, risk factors for anaemia and research gaps identified.

## 3. Results

### 3.1. Study Results

**Figure 1:**
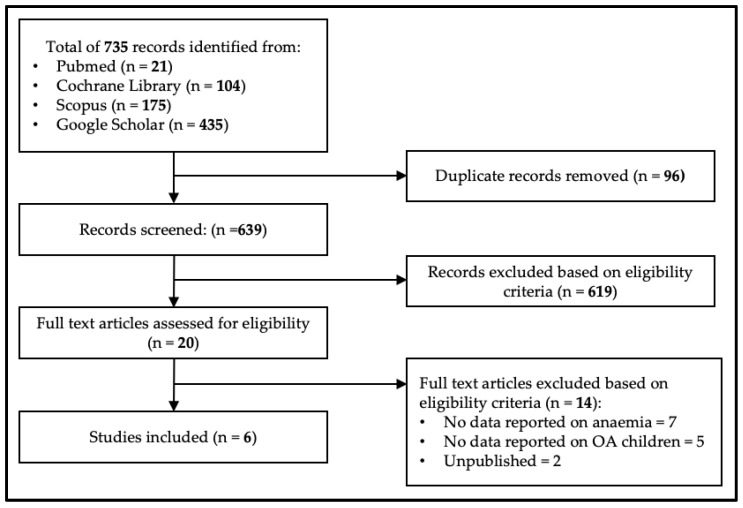
Prisma flow diagram.

This study search yielded 735 articles from the electronic databases (Pubmed = 21, Cochrane Library = 104, Scopus = 175 and Google Scholar = 435). No additional articles were identified from other sources. After removing 96 duplicate articles, another 619 articles were removed from title and abstract screening. Then, the full texts of twenty shortlisted articles were reviewed for inclusion, and fourteen articles were excluded with reasons. Subsequently, six articles that met the eligibility criteria were included in this review. Figure 1 summarises the study identification process.

### 3.2. Characteristics of Included Studies

**Figure 2:**
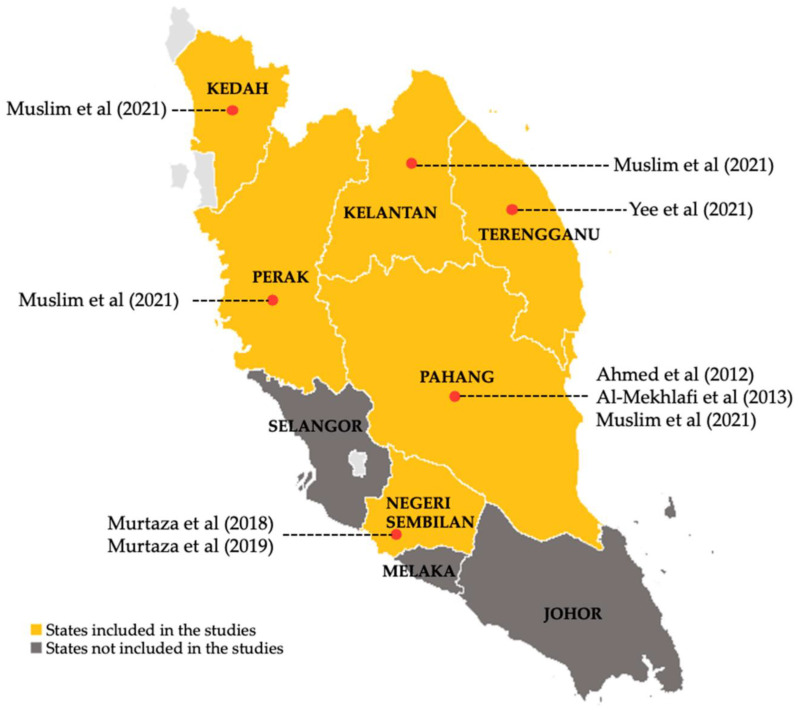
Locality of Orang Asli children population participated in the studies [24,25,26,27,28,29].

**Figure 3:**
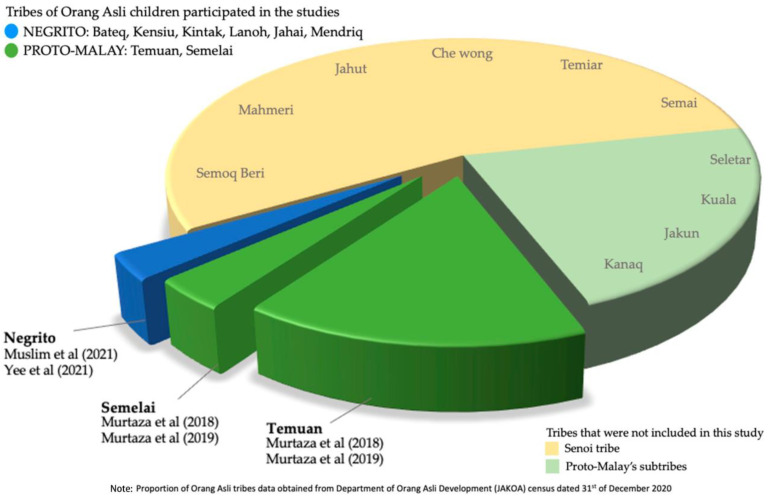
Tribes of Orang Asli children participated in the studies [25,27,28,29].

Among the six studies included in this review, only one was a randomised controlled trial study [24], while the rest were cross-sectional surveys. Orang Asli children involved in these studies were from six states in Peninsular Malaysia: Kedah [25], Perak [25], Pahang [24,25,26], Negeri Sembilan [27,28], Kelantan [25] and Terengganu [29] (Figure 2). As for the OA subtribes of the children in this study, there were two subtribes from the Proto-Malay tribe (Semelai and Temuan) and all the subtribes of the Negrito tribe (Figure 3). Furthermore, the number of participants in each study ranged from 77 to 343 children, and most of the studies, except for one [25], exclusively focused on the OA children population. Among all the included studies, three studies [24,25,26] measured anaemia as the primary outcome. The age group of OA children reported for anaemia prevalence in these studies ranged from two to thirteen years old. Apart from measuring anaemia among OA children, some studies also analysed other conditions associated with anaemia. Four studies reported soil-transmitted helminth infection [25,26,27,28], while one reported iron deficiency [24]. Characteristics of the included studies are summarised in Table 1.

### 3.3. Prevalence of Anaemia among Orang Asli Children in Malaysia

The overall prevalence of anaemia among OA children in Malaysia ranged from 21.6 to 80.0%. Detailing prevalence by different age groups, two studies (N = 533) reported that the prevalence of anaemia among those 2–6 years of age was close to 22.0%, but one study (Muslim et al., N = 32) reported a significantly higher prevalence of 80.0%. Similarly, for children in the 6–13 years age group, two studies (N = 504) reported the prevalence of anaemia ranging from 41.0 to 48.5% while one study (Muslim et al., N = 76) reported a higher prevalence of 70.4%. The highest prevalence of anaemia among OA children was observed among the Negrito tribe, while the lowest was among the Proto-Malay tribe (Semelai and Temuan) (Table 1). The prevalence of IDA among OA children was 34.0%, as reported by one study [24] in this review. A more recent study in this review reported that the prevalence of anaemia was higher among female OA children [29], contrary to the previous study [26]. The prevalence of mild anaemia was 12.9%, while moderate and severe anaemia among OA children ranged from 3.0 to 8.7% [25,27]. One study reported on locality-specific anaemia prevalence [25], which revealed a higher prevalence of anaemia of 82.4% among OA children aged 2–6 years living within inland forest territories (IJV) compared to 78.3% among those living in resettlement near town peripheries (RPS). On the contrary, a higher prevalence of anaemia of 75.0% was observed among OA children aged 7–12 years living in resettlement near town peripheries compared to 57.1% among those living within inland forest territories [25]. Anaemia-specific prevalence of OA children is summarised in Table 2.

### 3.4. Risk Factors of Anaemia among Orang Asli Children in Malaysia

Two studies analysed the risk factors of anaemia among OA people; however, only one was included in this review analysis [26]. The study found that haemoglobin was significantly lower in OA children aged less than ten years old and in those with moderate to heavy Ascaris infections with an adjusted odds ratio of 2.11 (95% CI 1.23, 3.63) and 2.05 (95% CI 1.12, 3.76), respectively (Table 1). The other study did not perform separate analyses for the OA children; hence the results were deemed irrelevant for this review purpose [25].

### 3.5. Research Gaps Identified

There was an absence of data from certain OA children from different demographics. First, OA children residing in Selangor, Melaka and Johor states were not included in this review (Figure 2). Next, OA children from ten subtribes were not reported in this review, involving all Senoi subtribes (Temiar, Che Wong, Mahmeri, Jahut, Semai and Semoq Beri) and four Proto-Malay subtribes (Kuala, Kanaq, Seletar, and Jakun) (Figure 3). Additionally, the OA children aged less than two years old and more than thirteen years old were not reported in this review. Otherwise, this review also found varying data reporting formats on anaemia between the studies, particularly the severity and gender-specific prevalence. Finally and above all, this review revealed the paucity of data on risk factors for anaemia among OA children in Malaysia at present.

## 4. Discussion

The emphasis on the review subject highlights the overall determinant of health inequities among indigenous people, which contribute to the burden of anaemia in the population. It is critical to note that this review contributes to sustainable development goals (SDGs) adopted by the Malaysian government from the United Nations (UN). Striving to reduce morbidity and mortality of anaemia among OA children contributes directly to SDGs (goal 3), specifically the neonatal mortality and under-five mortality rate [30]. Additionally, obtaining universal health coverage is the primary goal in the SDGs to address health disparities among marginalised groups, such as indigenous people, as it provides a link between equitable social and economic growth [31]. In this review, we aimed to summarise the existing data on anaemia among OA children to identify the prevalence and risk factors of anaemia and the gaps in anaemia research among OA children in Malaysia between 2012 and 2022. We identified six studies based on eligibility criteria, and the results uncovered significant findings from the currently available research evidence. First, we discovered little research focus among the children, resulting in limited inclusivity of children from all age groups and subtribes. Considering the large proportion of OA children accounting for up to 35% (77,000 children) of the total OA population [32], the participation of OA children in the included studies is still lacking. This is also supported by the absence of data on anaemia among Senoi tribe children, which is the largest tribe comprise of 55.1% of overall OA population [13]. Furthermore, we also found varying data reporting formats on anaemia between the studies, likely due to the different predetermined primary outcomes. As a result, some essential data on anaemia, like the severity prevalence, were missed in the publications. It is important to note that severe anaemia carries the highest morbidity and mortality among children [33].

Next, the results from our review accentuated the magnitude of anaemia among OA children based on the available evidence. The WHO classified the public health significance of anaemia based on population prevalence as normal (≤4.9%), mild (5–19.9%), moderate (20–39.9%) and severe (≥40%). According to this, current data on the prevalence of anaemia among OA children would be classified as a moderate to severe problem. Our results revealed that the prevalence of anaemia among OA children in both age groups of 2–6 years and 6–13 years was significantly higher than the major children population in Malaysia. According to the data from a nationwide cross-sectional study conducted among children in Malaysia, Poh et al. reported the prevalence of anaemia was higher among younger children aged 4–6 (11.3 to 17.6%) years old compared to older children aged 7–12 years old (3.6 to 5.1%) [8]. A similar trend was observed in our data obtained from the Muslim et al. study, which included OA children of different age groups. This trend is also consistent with global estimates of anaemia by the WHO which show higher prevalence of anaemia among preschool-aged children at 47.4% compared to school-aged children at 25.4% [1]. In comparison, the current prevalence of anaemia among OA children in Malaysia is also higher when compared to indigenous children in other Southeast Asian countries. For instance, two studies in Chiang Mai Province in Thailand reported that the prevalence of anaemia among Karen hill-tribe children was 19.8% in the 1–6 years age group [34] and 8.5% in the 8–11 years age group [35]. Meanwhile, the National Nutrition Survey in the Philippines reported the prevalence of anaemia among indigenous people children was 16.6% in the age group from 6 months to 5 years of age and 12.3% in the 6–12 years age group [36]. On another note, the significantly high prevalence of anaemia reported in our study by Muslim et al. conducted among the Negrito tribe, compared to the rest of included studies, is particularly interesting. Despite the arguably low number of respondents included in that study, it is important to note that Negrito tribe only comprise of 2.94% of overall OA population [13]. Thus, this data may suggest the existence of different level of socio-economic background and anaemia risk factors among the OA tribes. Studies on nutritional status among OA children of different tribes also showed majority of the indicators of malnutrition were more prevalent among the Negrito tribe [25,27,37]. Otherwise, another important factor to consider is that OA people who are living in the remote areas can either be living in a town periphery or deep in the jungle. According to JAKOA, about 61% of OA people have been relocated to town peripheries under the resettlement plans while another 38% of OA people are still living deep in the jungle with a small portion of them still being semi-nomadic, especially the Negrito tribe [14,38]. Those who have been relocated to town peripheries are expected to have better living conditions as they were provided with better necessities such as housing, school, electricity and a water supply by the government.

Our results also revealed the dearth of data on risk factors for anaemia among OA children in Malaysia. While aetiologies of anaemia are multifaceted, the significant causes of anaemia among the indigenous population are nutritional deficiency, particularly iron deficiency, malaria and helminth infection [15]. To date, there are several studies conducted on anaemia among indigenous children in other countries [17,18,39,40,41], while regrettably, our review only found a couple of anaemia risk factors reported by one study. Examples of anaemia risk factors identified among indigenous children to date were children’s age, gender, prolonged breastfeeding, maternal age, maternal education, household income, household condition, infection and more. On the other hand, previous local data published more than a decade ago identified some risk factors for anaemia among OA children, including gender, being underweight, stunting, severe trichuriasis, low level of mother’s education and having a working mother [19,20]. Therefore, it is clear that there is a huge gap in the current data on anaemia risk factors among OA children in Malaysia, and it should be addressed in future studies.

Indigenous children, including the OA children in Malaysia, are at risk for anaemia due to the vast disparities in social determinants of health, such as poverty, food insecurity, living environment and access to health services, compared to the general population [15]. A recent study revealed that the nutritional status of the OA is typically low among women and children population [42]. In response to this conundrum, the Ministry of Health Malaysia has made multiple efforts, including several nutritional programmes for the OA population, for instance, the Community Feeding Programme and the monthly food baskets under Rehabilitation Program for Malnourished Children [43]. In addition, nutritional status and parasitic infections among the OA population in Malaysia have gained more research focus, evident by increasing numbers of published articles over the last decade [44,45,46,47,48,49,50,51]. However, most of the available studies focused on nutritional status, dietary assessment and soil-transmitted helminth infection, while only a few reported on anaemia among OA people, especially children. This may be due to the hefty cost of including bio-specimen analysis in the study, limited access in some remote areas or difficulty obtaining voluntary participation from OA people, especially when an invasive investigation towards their children is involved.

### 4.1. Strength and Limitation

To the best of our knowledge, this review is the first to summarise available data on anaemia among OA children in Malaysia. We performed thorough search, carefully chose the studies and systematically extracted the data to obtain information on the prevalence, severity and risk factors of anaemia among OA children. However, we acknowledge several limitations in this review. First, the small number of included studies limits the data obtained in this review, primarily the risk factors of anaemia among OA children. Moreover, we did not perform bias and quality assessments of the included studies. Additionally, we excluded unpublished papers and restricted our review to only the Malay and English languages.

### 4.2. Recommendations for Future Research

The results from our review urge more recognition of anaemia among OA children as an essential public health concern in Malaysia. Our review results also urge for a more comprehensive epidemiological study in the future to reduce morbidity and mortality among OA children effectively. Therefore, future studies are advised to focus more on aetiologies of anaemia and associated risk factors among OA children to assist policymakers in devising effective programmes specific to OA children. Data on anaemia among OA children is extremely valuable due to the limited availability; thus, future studies are encouraged to perform in-depth analysis and reporting of data on anaemia obtained from OA children.

## 5. Conclusions

In Malaysia, the prevalence of anaemia among OA children constitutes a moderate to serious public health issue. Despite multiple efforts taken by the Ministry of Health Malaysia, anaemia continues to be a burden among the OA population, primarily the children. A huge research gap identified in this review predominantly concerning the associated risk factors urges for more comprehensive future research to obtain more accurate data representing the OA children population in Malaysia. This data will assist policymakers in devising more effective national prevention strategies to improve morbidity and mortality among OA children in the future.

## Figures and Tables

**Table 1:** Overall characteristics of included studies on anaemia among OA children in Malaysia, 2012–2022.

Author(s) (Year)	Primary Objective	Study Design	Sample Size	Age (Years)	Condition	Prevalence of Anaemia (%)	Risk Factors for Anaemia	AOR (95% CI), *p*-Value
Ahmed et al. [26] (2012)	To determine the current prevalence of anaemia and malnutrition	Cross-sectional	254	6–13	Anaemia, STH	41.0	Age (<10 years) Ascaris Infections (Moderate-to-heavy)	2.11 (1.23, 3.63) *p* = 0.007 2.05 (1.12, 3.76) *p* = 0.021
Al-Mekhlafi et al. [24] (2013)	To investigate the effect of vitamin A supplementation on iron status indices and IDA status among OA primary schoolchildren in rural Malaysia	Randomised Controlled Trial	250	7–12	Anaemia, IDA	48.5	NA	NA
Murtaza et al. [27] (2018)	To identify the factors associated with stunting among OA preschool children in Negeri Sembilan	Cross-sectional	264	2–6	Anaemia, STH	21.6	NA	NA
Murtaza et al. [28] 2019)	To study the determining factors associated with OA children’s cognitive performance	Cross-sectional	269	2–6	Anaemia, STH	21.7	NA	NA
Yee et al. [29] (2021)	To determine nutritional status by anthropometric measurements and biochemical assessments	Cross-sectional	77	7–12	Anaemia	61.6	NA	NA
Muslim et al. [25] (2021)	To compare the current status of malnutrition, anaemia and their associations with STH infections and other selected variables between IJV and RPS	Cross-sectional	108	2–12	Anaemia, STH	Age 2–6: 80.0 Age 7–12: 70.4	NA	NA

OA, Orang Asli; AOR, adjusted odds ratio; STH, soil-transmitted helminth; NA, not available; IDA, iron deficiency anaemia.

**Table 2:** Anaemia-specific prevalence among OA children in Malaysia.

Author(s) (Year)	Gender (%)		Anaemia Severity Classification (%)			IDA (%)	STH Infections (%)		Locality (%)	
	Male	Female	Mild	Moderate	Severe		Negative-to-Light	Moderate-to-Heavy	IJV	RPS
Ahmed et al. [26] (2012)	45.1	37.1	NA	NA	NA	NA	34.0%	44.6%	NA	NA
Al-Mekhlafi et al. [24] (2014)	NA	NA	NA	NA	NA	34.0	NA	NA	NA	NA
Murtaza et al. [27] (2018)	NA	NA	12.9	8.7		NA	NA	NA	NA	NA
Murtaza et al. [28] 2019)	NA	NA	NA	NA	NA	NA	NA	NA	NA	NA
Yee et al. [29] (2021)	59.5	63.9	NA	NA	NA	NA	NA	NA	NA	NA
Muslim et al. [25] (2021)	NA	NA	NA	NA	3.0	NA	NA	NA	Age 2–6: 82.4 Age 7–12: 57.1	Age 2–6: 78.3 Age 7–12: 75.0

STH, soil-transmitted helminth; IDA, iron deficiency anaemia; NA, not available; IJV, inland jungle village; RPS, resettlement plan scheme.

## Data Availability

Not applicable.

## Supplementary Materials

The following supporting information can be downloaded at: https://www.mdpi.com/article/10.3390/nu15061493/s1, Table S1: PRISMA-ScR checklist. Figure S1: The Detailed Search Strategy. Reference [22] was cited in the supplementary materials.
