# Supplementary material for: Prevalence and Risk Factors of Anaemia among Orang Asli Children in Malaysia: A Scoping Review
Source: Nutrients. 2023 Mar 20;15(6):1493. doi: 10.3390/nu15061493 (PMC10053598; doi:10.3390/nu15061493)
Supplement: Supplementary file 1 [file nutrients-15-01493-s001.zip › Supplementary Figure S1.pdf]

Supplementary Figure S1: The Detailed Search Strategy

PUBMED

("anemia"[MeSH Terms] OR anaemia OR haemoglobin OR “iron-deficiency anaemia”) AND (aborigin* OR indigenous OR “orang asli” OR negrito OR semang OR senoi OR “aboriginal malay” OR “melayu proto”) AND (child* OR boy OR girl OR “school children” OR adolescent OR teen* OR paediatric OR neonat OR infant OR newborn OR toddler)

COCHRANE LIBRARY

#1	(MeSH descriptor: [] explode all trees and with qualifier(s): [epidemiology - EP]
#2	anaemia OR haemoglobin OR "iron-deficiency anaemia" with Cochrane Library publication date Between Jan 2012 and Mar 2022
#3	aborigin* OR indigenous OR “orang asli” OR negrito OR semang OR senoi OR “aboriginal malay” OR “melayu proto” with Cochrane Library publication date Between Jan 2012 and Mar 2022
#4	child* OR boy OR girl OR “school children” OR adolescent OR teen* OR paediatric OR neonat OR infant OR newborn OR toddler
#5	#1 OR #2
#6	#5 AND #3 AND #4

SCOPUS

(anaemia OR haemoglobin OR "iron-deficiency anaemia") AND (aborigin* OR indigenous OR "orang asli" OR negrito OR semang OR senoi OR "aboriginal malay" OR "melayu proto") AND (child* OR boy OR girl OR "school children" OR adolescent OR teen* OR paediatric OR neonat* OR infant OR newborn OR toddler) AND (LIMIT-TO (PUBYEAR , 2022) OR LIMIT-TO (PUBYEAR , 2021) OR LIMIT-TO (PUBYEAR , 2020) OR LIMIT-TO (PUBYEAR , 2019) OR LIMIT-TO (PUBYEAR , 2018) OR LIMIT-TO (PUBYEAR , 2017) OR LIMIT-TO (PUBYEAR , 2016) OR LIMIT-TO (PUBYEAR , 2015) OR LIMIT-TO (PUBYEAR , 2014) OR LIMIT-TO (PUBYEAR , 2013) OR LIMIT-TO (PUBYEAR , 2012)) AND (LIMIT-TO (AFFILCOUNTRY , "Malaysia"))

GOOGLE SCHOLAR

"anaemia" "orang asli" "children" in malaysia
