# Supplementary material for: Prevalence and Risk Factors of Anaemia among Orang Asli Children in Malaysia: A Scoping Review
Source: Nutrients. 2023 Mar 20;15(6):1493. doi: 10.3390/nu15061493 (PMC10053598; doi:10.3390/nu15061493)
Supplement: Supplementary file 1 [file nutrients-15-01493-s001.zip › Supplementary Table S1.pdf]

Supplementary Table S1: PRISMA-ScR checklist

SECTION	ITEM	PRISMA-ScR CHECKLIST ITEM	REPORTED ON PAGE #
**TITLE**			
Title	1	Identify the report as a scoping review.	1
**ABSTRACT**			
Structured summary	2	Provide a structured summary that includes (as applicable): background, objectives, eligibility criteria,sources of evidence, charting methods, results, and conclusions that relate to the review questions andobjectives.	1
**INTRODUCTION**			
Rationale	3	Describe the rationale for the review in the context ofwhat is already known. Explain why the review questions/objectives lend themselves to a scopingreview approach.	2
Objectives	4	Provide an explicit statement of the questions and objectives being addressed with reference to their key elements (e.g., population or participants, concepts, andcontext) or other relevant key elements used to conceptualize the review questions and/or objectives.	2
**METHODS**			
Protocol and registration	5	Indicate whether a review protocol exists; state if and where it can be accessed (e.g., a Web address); and ifavailable, provide registration information, including theregistration number.	NA
Eligibility criteria	6	Specify characteristics of the sources of evidence usedas eligibility criteria (e.g., years considered, language, and publication status), and provide a rationale.	3
Information sources*	7	Describe all information sources in the search (e.g., databases with dates of coverage and contact with authors to identify additional sources), as well as thedate the most recent search was executed.	3
Search	8	Present the full electronic search strategy for at least 1 database, including any limits used, such that it could berepeated.	3
Selection of sources of evidence†	9	State the process for selecting sources of evidence (i.e.,screening and eligibility) included in the scoping review.	3
Data charting process‡	10	Describe the methods of charting data from the includedsources of evidence (e.g., calibrated forms or forms thathave been tested by the team before their use, and whether data charting was done independently or in duplicate) and any processes for obtaining and confirming data from investigators.	3
Data items	11	List and define all variables for which data were soughtand any assumptions and simplifications made.	3
Critical appraisal of individual sources of evidence§	12	If done, provide a rationale for conducting a critical appraisal of included sources of evidence; describe the methods used and how this information was used in anydata synthesis (if appropriate).	NA
Synthesis of results	13	Describe the methods of handling and summarizing thedata that were charted.	3, 4 & 5

* Where *sources of evidence* (see second footnote) are compiled from, such as bibliographic databases, social media platforms, and Web sites. † A more inclusive/heterogeneous term used to account for the different types of evidence or data sources (e.g., quantitative and/or qualitative research, expert opinion, and policy documents) that may be eligible in a scoping review as opposed to only studies. This is not to be confused with *information sources* (see first footnote).

‡ The frameworks by Arksey and O'Malley (6) and Levac and colleagues (7) and the JBI guidance (4, 5) refer to the process of data extraction in a scoping review as data charting.

§ The process of systematically examining research evidence to assess its validity, results, and relevance before using it to inform a decision. This term is used for items 12 and 19 instead of "risk of bias" (which is more applicable to systematic reviews of interventions) to include and acknowledge the various sources of evidence that may be used in a scoping review (e.g., quantitative and/or qualitative research, expert opinion, and policy documents).

From: Tricco AC, Lillie E, Zarin W, O'Brien KK, Colquhoun H, Levac D, et al. PRISMA Extension for Scoping Reviews (PRISMAScR): Checklist and Explanation. Ann Intern Med. 2018;169:467–473. doi: 10.7326/M18-0850.
